# *In vitro* Characterization of Fitness and Convalescent Antibody Neutralization of SARS-CoV-2 Cluster 5 Variant Emerging in Mink at Danish Farms

**DOI:** 10.3389/fmicb.2021.698944

**Published:** 2021-06-25

**Authors:** Ria Lassaunière, Jannik Fonager, Morten Rasmussen, Anders Frische, Charlotta Polacek, Thomas Bruun Rasmussen, Louise Lohse, Graham J. Belsham, Alexander Underwood, Anni Assing Winckelmann, Signe Bollerup, Jens Bukh, Nina Weis, Susanne Gjørup Sækmose, Bitten Aagaard, Alonzo Alfaro-Núñez, Kåre Mølbak, Anette Bøtner, Anders Fomsgaard

**Affiliations:** ^1^Virus and Microbiological Special Diagnostics, Statens Serum Institut, Copenhagen, Denmark; ^2^Department of Veterinary and Animal Sciences, Faculty of Health Sciences, University of Copenhagen, Copenhagen, Denmark; ^3^Copenhagen Hepatitis C Program (CO-HEP), Department of Immunology and Microbiology, Faculty of Health and Medical Sciences, University of Copenhagen, Copenhagen, Denmark; ^4^Department of Infectious Diseases, Copenhagen University Hospital, Hvidovre, Copenhagen, Denmark; ^5^Department of Clinical Medicine, Faculty of Health and Medical Sciences, University of Copenhagen, Copenhagen, Denmark; ^6^Department of Clinical Immunology, Zealand University Hospital, Naestved, Denmark; ^7^Department of Clinical Immunology, Aalborg University Hospital, Aalborg, Denmark; ^8^Division of Infectious Diseases Preparedness, Statens Serum Institut, Copenhagen, Denmark

**Keywords:** SARS-CoV-2, COVID-19, mink, coronavirus, virus neutralization

## Abstract

In addition to humans, severe acute respiratory syndrome coronavirus 2 (SARS-CoV-2) can transmit to animals that include hamsters, cats, dogs, mink, ferrets, tigers, lions, cynomolgus macaques, rhesus macaques, and treeshrew. Among these, mink are particularly susceptible. Indeed, 10 countries in Europe and North America reported SARS-CoV-2 infection among mink on fur farms. In Denmark, SARS-CoV-2 spread rapidly among mink farms and spilled-over back into humans, acquiring mutations/deletions with unknown consequences for virulence and antigenicity. Here we describe a mink-associated SARS-CoV-2 variant (Cluster 5) characterized by 11 amino acid substitutions and four amino acid deletions relative to Wuhan-Hu-1. Temporal virus titration, together with genomic and subgenomic viral RNA quantitation, demonstrated a modest *in vitro* fitness attenuation of the Cluster 5 virus in the Vero-E6 cell line. Potential alterations in antigenicity conferred by amino acid changes in the spike protein that include three substitutions (Y453F, I692V, and M1229I) and a loss of two amino acid residues 69 and 70 (ΔH69/V70), were evaluated in a virus microneutralization assay. Compared to a reference strain, the Cluster 5 variant showed reduced neutralization in a proportion of convalescent human COVID-19 samples. The findings underscore the need for active surveillance SARS-CoV-2 infection and virus evolution in susceptible animal hosts.

## Introduction

Severe acute respiratory syndrome coronavirus 2 (SARS-CoV-2) emerged in December 2019 in Wuhan, China. It has since caused a pandemic that has resulted in more than 140 million confirmed cases of coronavirus disease 2019 (COVID-19) and over 3 million deaths by 18 April 2021 (World Health Organization, 2021). Throughout the pandemic, there has been the threat that new variants with increased transmissibility, pathogenesis, or immune evasion may arise. Indeed, in humans, SARS-CoV-2 variants of concern were described since October 2020 in the United Kingdom (lineage B.1.1.7), Brazil and Japan (P.1), United States of America (B.1.427 and B.1.429), and South Africa (B.1.351) (Public Health England, 2020; Faria et al., 2021; Tegally et al., 2021). The variants display different degrees of enhanced transmissibility (Davies et al., 2021; Deng et al., 2021; Faria et al., 2021; Pearson et al., 2021) and, with the exception of B.1.1.7, reduced neutralization by convalescent sera, post-vaccination sera and/or monoclonal antibody therapeutics (Deng et al., 2021; Food and Drug Administration, 2021; Madhi et al., 2021; Wang et al., 2021; Wibmer et al., 2021). Virus evolution in other hosts may similarly lead to new SARS-CoV-2 variants.

Experimental infection and reports of human-to-animal transmission identified animal species that are susceptible for SARS-CoV-2 infection. These include hamsters, cats, dogs, mink, ferrets, tigers, lions, cynomolgus macaques, rhesus macaques, and treeshrew (Mahdy et al., 2020). Among these, the largest reported outbreaks of SARS-CoV-2 occurred among production mink on fur farms (Oreshkova et al., 2020; Boklund et al., 2021; Hammer et al., 2021; Oude Munnink et al., 2021). SARS-CoV-2 has been detected in farmed mink in Canada, Denmark, France, Greece, Italy, Lithuania, the Netherlands, Spain, Sweden, and the United States of America (Food and Agriculture Organization, 2021). Within Denmark, mink at 290 of 1,147 (25%) farms tested positive for SARS-CoV-2 between June and November 2020 (Boklund et al., 2021), corresponding to approximately 3–4 million infected animals compared with less than an estimated total of 300,000 infected people in Denmark (Larsen et al., 2021).

As observed in humans, sustained infection and transmission of SARS-CoV-2 in a new animal host, such as mink, affords an opportunity for evolutionary changes in the virus that may result in variants with potential consequences for transmissibility, pathogenicity and immune evasion in humans. In Denmark, the passage of SARS-CoV-2 through a myriad of mink lead to the accumulation of nucleotide changes in the viral genome (Hammer et al., 2021; Larsen et al., 2021). Despite the mutations likely favoring virus replication in the mustelid host, mink-associated SARS-CoV-2 variants successfully established infections in farm workers and spread to surrounding communities (Larsen et al., 2021). An estimated 4,000 individuals were infected with mink-associated SARS-CoV-2 variants in Denmark (Larsen et al., 2021). Thus, the consequences of the mutations on SARS-CoV-2 infectivity, virulence, and antigenicity warranted investigation.

## Results

### Accumulation of Amino Acid Changes in the Spike Protein of Mink-Associated SARS-CoV-2

**FIGURE 1:**
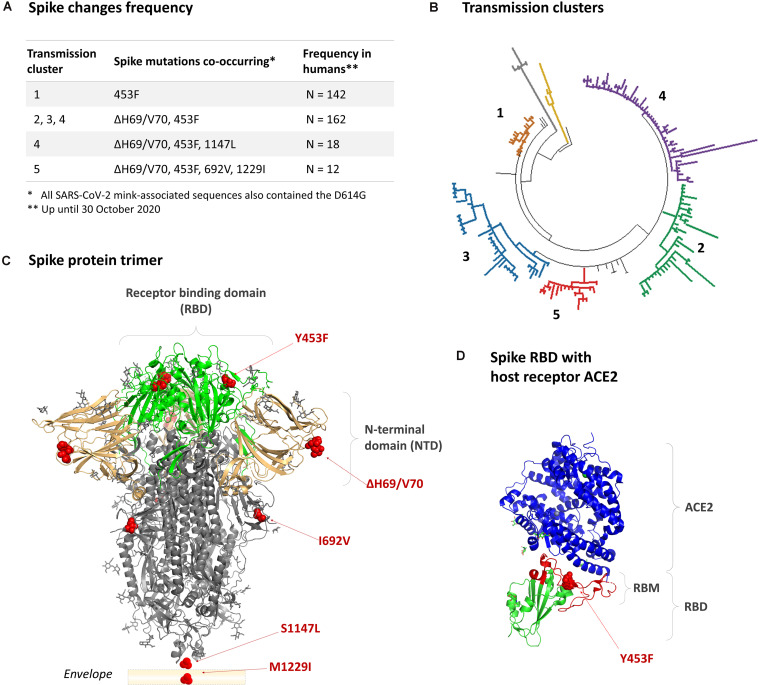
The mink-associated mutations in the SARS-CoV-2 spike protein. **(A)** The combination and frequency of mink-associated spike changes detected in SARS-CoV-2 infected humans up until 30th October 2020. **(B)** Phylogenetic grouping of mink-associated variant transmission Clusters 1–5 (lineage B.1.1.298). **(C)** The crystal structure of a closed pre-fusion spike trimer [PDB: 6ZGE]. The positions of amino acid changes are indicated with red spheres. The receptor binding domain (RBD) is indicated in green, the N-terminal domain (NTD) in beige, and the S2 domain in gray. The regions encompassing the S1147L and M1229I substitutions are not within the crystal structure; however, their relative positions are indicated. **(D)** The location of the Y453F substitution in the receptor binding domain complexed with a host ACE2 receptor (blue) [PBD: 6LZG].

In Denmark, SARS-CoV-2 variants co-circulating in mink and humans collectively acquired at least 35 different amino acid changes in the spike protein (Larsen et al., 2021). These SARS-CoV-2 variants belong to lineage B.1.1.298. The receptor binding domain (RBD) substitution Y453F, also observed among Dutch farmed mink (Oude Munnink et al., 2021), appeared in the first transmission Cluster in June (Cluster 1; Figure 1; Hammer et al., 2021). A two-amino acid residue deletion (ΔH69/V70) in the N-terminal domain appeared together with the Y453F in August 2020 and occurred in the subsequent Clusters 2, 3, and 4 (Figure 1). A new variant, termed Cluster 5, with two additional amino acid substitutions, i.e., I692V downstream of the transmembrane protease serine 2 (TMPRSS2)/furin cleavage site and M1229I within the transmembrane domain, was identified in September 2020 on 5 mink farms and in 12 human cases (age: 7–79 years; symptoms: asymptomatic to mild). Given the potential of this variant to spread among humans, as observed for other mink-associated variants (Hammer et al., 2021; Oude Munnink et al., 2021), and an increased risk of antigenic alterations with the multiple spike changes, it was deemed necessary to do a rapid evaluation of this “Cluster 5” variant *in vitro*. To aid timely public health responses, Statens Serum Institut released a preliminary report on 10 November 2020 (Lassaunière et al., 2020). A more detailed evaluation of this Cluster 5 variant and its *in vitro* fitness and neutralization potential is presented here.

### Cluster 5 Variant Growth Kinetics in a Vero-E6 Cell Culture Model

**FIGURE 2:**
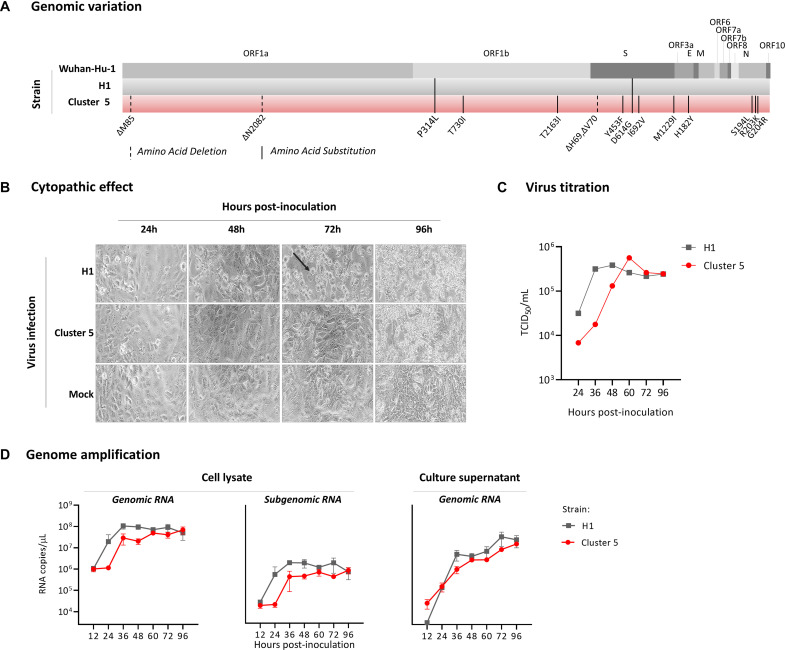
Genomic characterization and growth kinetics of the SARS-CoV-2 Cluster 5 variant in Vero-E6 cells. **(A)** Amino acid changes identified in the SARS-CoV-2 Cluster 5 variant and a Danish SARS-CoV-2 strain (H1) isolated in March 2020 relative to the reference strain Wuhan-Hu-1. **(B)** Temporal cytopathic effect of Vero-E6 cells following infection with the two virus strains at a multiplicity of infection (MOI) of 0.01. The arrow indicates the characteristic rounding of SARS-CoV-2 infected cells. **(C)** Temporal increase in H1 and Cluster 5 virus levels following inoculation of Vero-E6 cells at an MOI of 0.01. At the indicated time points, the virus titre was determined in a SARS-CoV-2-specific anti-nucleocapsid protein ELISA and the TCID_50_/mL calculated using the Reed and Muench method. **(D)** Quantitation of SARS-CoV-2 E gene genomic and subgenomic RNA in infected Vero-E6 cells and cell culture supernatant. Cells were infected at a multiplicity of infection of 0.0125 for 1 h, washed three times to remove all inoculum, overlaid with culture media, and cells and culture supernatant harvested at the indicated time points.

The Cluster 5 variant was isolated from a human throat swab sample. This variant virus strain, named hCoV-19/Denmark/DCGC-3024/2020 (GISAID EPI_ISL_616802; passage 0), has 11 amino acid substitutions and 4 amino acid deletions relative to the reference strain Wuhan-Hu-1 (Figure 2A). Of these, three amino acid substitutions (Y453F, I692V, and M1229I) and a two amino acid deletion (ΔH69/V70) occur in the spike protein (Figure 1C and Supplementary Figure 1 relative to circulating variants of concern). For characterization, the virus was passaged twice in Vero-E6 cells. Whole genome sequencing confirmed that the virus sequence remained unaltered from the original clinical sample. In Vero-E6 cells, the novel variant induced a delayed and less pronounced cytopathic effect compared to a representative SARS-CoV-2 isolate (Figure 2B). In agreement, using a quantitative anti-SARS-CoV-2 nucleocapsid protein ELISA as proxy for measuring virus levels, a delayed increase in virus concentration (TCID_50_/mL) was measured for the Cluster 5 variant compared to an early epidemic Danish SARS-CoV-2 isolate (Figure 2C) and other isolates (data not shown). SARS-CoV-2 E gene genomic and subgenomic RNA measurements for the Cluster 5 virus were also notably lower at 24 h post-inoculation compared to the early pandemic isolate (Figure 2D). At 96 h, both viruses had the same TCID_50_/mL and RNA levels. The ability to replicate to high viral titres is consistent with high levels of the virus variant detected in the clinical throat swabs, with a median diagnostic qPCR assay cycle threshold (Ct) of 23.7 (range: 20–35).

### Antigenicity of the Cluster 5 Variant

**FIGURE 3:**
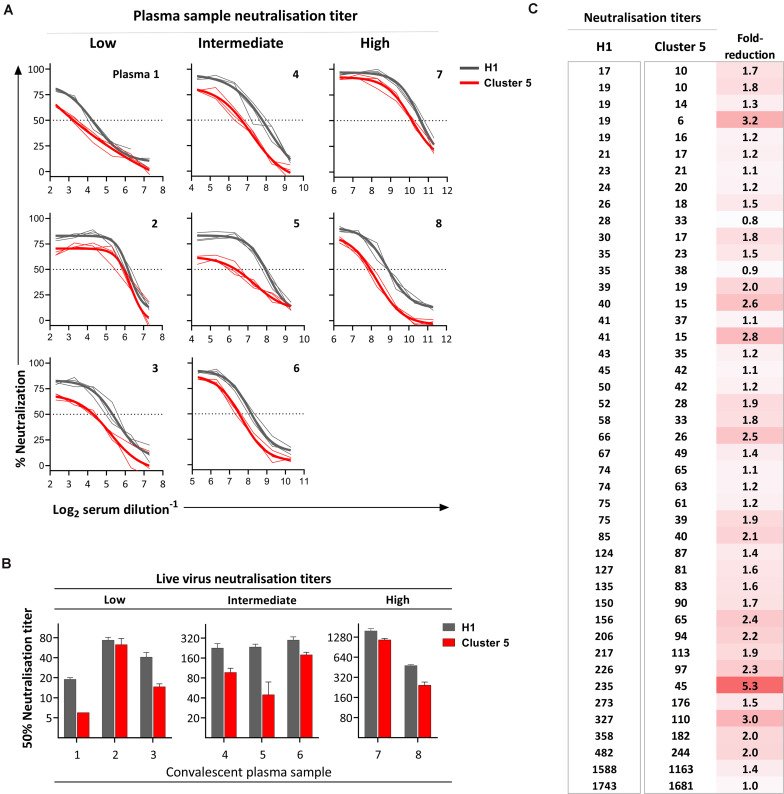
Neutralization of the SARS-CoV-2 Cluster 5 virus relative to an early epidemic SARS-CoV-2 virus (H1). In all instances, neutralization was evaluated using live SARS-CoV-2 viruses isolated from clinical samples. **(A)** Neutralization activity of twofold serial diluted convalescent COVID-19 plasma was tested in triplicate with 300× TCID_50_ Cluster 5 or H1 virus. Thick lines represent the four-parameter titration curve calculated from the mean of triplicate measurements; thin lines represent each individual replicate. **(B)** The relative neutralization titres measured at 24 h post-inoculation for plasma samples for the individuals in **(A)** calculated as the interpolation of the four-parameter titration curve with the 50% cut-off value and presented as the reciprocal serum dilution. Bars represent the mean titre of triplicate measurements with the standard deviation. **(C)** Neutralization titres determined for 44 convalescent plasma from non-hospitalized individuals with PCR confirmed SARS-CoV-2 infection. Each line represents an individual plasma sample ranked by titre against the early pandemic strain.

To evaluate the effect of the mink-associated SARS-CoV-2 spike mutants on antigenicity, the neutralizing activity of a small selection of COVID-19 convalescent plasma samples with low (1:20–1:80), intermediate (1:160–1:320), and high (>1:320) neutralization titres were initially evaluated against Cluster 5 variant relative to an early pandemic SARS-CoV-2 isolate (H1) (Figures 3A,B). The convalescent plasma samples were collected from individuals who had documented SARS-CoV-2 infections at the beginning of the Danish epidemic prior to the mink outbreaks and who lived in the Capital Region of Denmark, which is geographically separated from the mink outbreaks in Northern Jutland. Eight out of the nine plasma samples evaluated in the preliminary report (Lassaunière et al., 2020) had sufficient material available for retesting. To ensure that the reduced neutralizing activity observed in the preliminary analysis was not an experimental artifact, all critical components in the microneutralization assay were changed to new lots or different equivalents. The repeat experiment confirmed the initially observed reduced neutralization activity against the Cluster 5 variant (Figures 3A,B). The Cluster 5 variant was further evaluated against an additional panel of convalescent plasma for a total 44 samples (Figure 3C). Overall, the neutralizing antibody titres were significantly lower for the Cluster 5 virus (median: 39.5, 95% confidence interval (95%CI): 28–63) compared with the early pandemic SARS-CoV-2 isolate (median: 62, 95%CI: 41–85; *P* < 0.05). In total, 13/44 samples (29.5%) had a ≥2-fold reduction in neutralization titre, 3/44 (6.8%) ≥3-fold reduction, and 1/44 (2.3%) ≥5-fold reduction. Only one plasma sample had a greater than fourfold reduction, a threshold set for neutralization resistance (Li et al., 2020).

## Discussion

Mink-associated SARS-CoV-2 variants of lineage B.1.1.298 contributed notably to the Danish human SARS-CoV-2 epidemic in 2020. In northern Denmark, mink variants constituted up to 53% of human COVID-19 cases in the weeks of 5–18 October 2020 (Larsen et al., 2021). In different transmission clusters, the mink SARS-CoV-2 variants continued to acquire additional amino acid changes in the spike protein, the primary target for neutralizing antibodies as well as elsewhere. The unhindered transmission and spread of mink-associated viruses in the community, together with the potential threat of new variants, necessitated an investigation of a newly emerged mink-associated variant (Cluster 5), which had four amino acid changes in the spike protein (ΔH69/V70, Y453F, I692V, and M1229I) in addition to D614G.

Virus isolation and limited passage in Vero-E6 cells demonstrated a modest fitness attenuation of the Cluster 5 virus in this cell line. The molecular mechanism underlying the slower growth remains to be determined. Potential causes may include: (i) reduced efficiency of viral entry due to the spike mutations; (ii) modulated viral replication resulting from amino acid changes and/or deletions in ORF1a (ΔM85 and ΔN2082) and ORF1b (P314L, T730I, and T2163I) that encode the viral replication and transcription complex (V’Kovski et al., 2021); or (iii) altered virus induced apoptosis mediated by ORF3a in the presence of the H182Y change (Ren et al., 2020). The observed *in vitro* fitness may be specific to Vero-E6 cells and, thus, warrants further investigations of the variant’s replication kinetics in different cell lines and primary cells.

In a virus microneutralization assay, we further demonstrated a reduced sensitivity of the Cluster 5 virus to neutralizing antibodies in a proportion of COVID-19 convalescent plasma samples. The observed variation in neutralization is likely attributable to the heterogeneity of polyclonal antibody responses of different individuals. In an independent study using pseudotyped virus neutralization assays, the Cluster 5 spike changes were also associated with a 3.4-fold decrease in neutralization titres in persons immunized with the BNT162b2 (Pfizer-BioNTech) mRNA vaccine (Garcia-Beltran et al., 2021), which is based on the Wuhan-Hu-1 reference strain. Neither study evaluated the contribution of the individual spike amino acid changes to the observed reduced neutralization; thus, precluding an assessment of the independent role of each amino acid change. However, the Y453F substitution and ΔH69/V70 were evaluated in other contexts.

The Y453F substitution in the receptor binding domain appeared independently in SARS-CoV-2 infected mink in the Netherlands and in Denmark. While infrequent in the Netherlands (4 out of 16 farms) (Oude Munnink et al., 2021), Y453F became an established mink-associated spike mutation in Denmark (Hammer et al., 2021). It occurred on multiple SARS-CoV-2 infected mink farms and frequently in the communities living near mink farms. Amino acid 453 directly interacts with the target host receptor angiotensin-converting enzyme 2 (ACE2) at amino acid 34, which differs between human (34H) and mink (34Y) (Damas et al., 2020; Wang et al., 2020). While Y453F is potentially an adaptation to the mink ACE2 receptor (van Dorp et al., 2020), it also increases affinity for human ACE2 (Starr et al., 2020; Motozono et al., 2021). Indeed, it has sporadically occurred in human cases without a known link to mink farms (European Centers for Disease Control, 2020). Y453F resides within an immunodominant HLA-A^∗^24:02 restricted epitope NF9 (Motozono et al., 2021). The NF9-Y453F epitope significantly reduces CD8^+^ T cell responses in HLA-A^∗^24-positive COVID-19 convalescent samples (Motozono et al., 2021). Moreover, the Y453F variant also arose as a neutralization resistance mutation in the presence of some therapeutic neutralizing antibodies, as demonstrated for Regeneron’s cocktail REGN10933 where Y453F reduced the neutralizing activity of an antibody by 29% (Baum et al., 2020; Starr et al., 2021).

Globally, the deletion ΔH69/V70 appeared in multiple different SARS-CoV-2 lineages, with at least three independent events (Kemp et al., 2020). Moreover, the ΔH69/V70 variant appeared under selective pressure during convalescent plasma therapy (Kemp et al., 2021). *In silico*, the deletion is predicted to alter the conformation of the N-terminal domain by pulling the protruding loop inwards (Kemp et al., 2020). *In vitro*, this deletion reduced the neutralizing activity of a non-RBD-targeting monoclonal COVA1-21 antibody by three to five fold (Kemp et al., 2021). Taken together, the Y453F and/or ΔH69/V70 may have contributed to the observed reduced neutralization sensitivity of the Cluster 5 variant for COVID-19 convalescent plasma samples.

In conclusion, the unbridled spread of SARS-CoV-2 through a myriad of mink has led to the accumulation of amino acid changes in the spike protein, the primary target of neutralizing antibodies. Ultimately, the combination of spike changes in the Cluster 5 variant conferred a degree of resistance to neutralizing antibodies in a proportion of convalescent COVID-19 people, which is in agreement with reduced titres observed for vaccinated individuals (Garcia-Beltran et al., 2021). Following the Cluster 5 discovery, a mink-associated SARS-CoV-2 variant with six spike protein changes appeared (L5F, ΔH69/V70, Y453F, D614G, N751Y, and C1250F). Thus, the virus continued to acquire spike protein changes through its passage in mink. SARS-CoV-2 infection in susceptible hosts should be controlled, where possible, and actively monitored through sequencing to identify emergent strains for characterization of virulence, transmission and neutralization resistance.

## Materials and Methods

### Primary Virus Isolation

In a 24-well plate, 5 × 10^4^ Vero-E6 cells were seeded per well and cultured overnight in culture medium [Dulbecco’s Modified Eagle Medium (DMEM), 10% fetal calf serum and 1% Penicillin/Streptomycin] at 37°C, 5% CO_2_. Prior to inoculation, the cell culture media was removed, the monolayer washed once with 1 mL phosphate buffered saline (PBS) and covered with 250 μL infection media (DMEM and 1% Penicillin/Streptomycin). Throat swabs submerged in PBS were used for the primary isolation of the SARS-CoV-2 cluster 5 variant and the early pandemic H1 virus. Both viruses were isolated from clinical samples obtained from people residing in Denmark. Each sample was passed through a 0.2 μm filter and added dropwise to an 80–90% confluent Vero-E6 cell monolayer. The cells were incubated with the inoculum for 1 h at 37°C, 5% CO_2_, followed by the addition of 1 mL growth media (DMEM, 1% Penicillin/Streptomycin, 10% fetal calf serum). The cell culture supernatant was harvested 72–96 h post-inoculation, clarified by centrifugation at 300 × *g* for 5 min, and stored at −80°C. All cell culture reagents were from Gibco, ThermoFisher Scientific, Waltham, Massachusetts, United States.

### Virus Passage

To expand the virus stocks, 1.5 × 10^6^ Vero-E6 cells were seeded in a 75 cm^2^ flask in culture medium and incubated overnight at 37°C, 5% CO_2_. The cell monolayer was washed once with 5 mL PBS and inoculated with 25 μL Passage 1 virus diluted in 2 mL DMEM. After a 1 h incubation with the inoculum at 37°C, 5% CO_2_, 10 mL virus growth media (DMEM with 5% fetal calf serum, 1% Penicillin/Streptomycin, and 10 mM HEPES buffer) was added and incubated at 37°C, 5% CO_2_. The virus was harvested 72 h later. The passage 2 virus stock was clarified by centrifugation at 300 × *g* for 5 min and the supernatant stored in single use aliquots at −80°C. All experiments were performed with the passage 2 stock. Whole genome sequencing of this stock confirmed that the virus sequence remained identical to that of the original clinical sample.

### Virus Titration

In a 96 well tissue culture plate, 1 × 10^4^ Vero-E6 cells were seeded per well and incubated overnight at 37°C, 5% CO_2_. Prior to inoculation, the cell monolayer was washed once with 200 μL PBS followed by the addition of 100 μL of SARS-CoV-2 virus serially diluted in virus diluent (DMEM, 1% Penicillin/Streptomycin, 2% bovine serum albumin, and 10 mM HEPES buffer). After a 1 h incubation at 37°C, 5% CO_2_, 100 μL growth media (DMEM, 10% fetal calf serum and 1% Penicillin/Streptomycin) was added to each well and incubated at 37°C, 5% CO_2_. Infection of wells was confirmed using an anti-SARS-CoV-2 nucleocapsid protein ELISA (described below) at 96 h post-inoculation where virus growth plateaued. Virus stock titres were calculated by the Reed and Muench method and expressed as the 50% tissue culture infectious dose per mL (TCID_50_/mL). The passage 2 virus stocks, stored in single use aliquots at −80°C, were titrated in triplicate. Each replicate titration assay tested quadruplicate serial dilutions of the H1 and Cluster 5 viruses, which were tested on the same 96-well tissue culture plate to eliminate inter-plate variation. The average titre of the triplicate measurements were used for inoculum determinations. On the day of each experiment that compared the virus strains, single use aliquots from the titrated stocks were diluted to the same virus concentration for equal inoculation.

### Anti-SARS-CoV-2 Nucleocapsid Protein ELISA

At the indicated time points, culture medium was removed from the infected Vero-E6 cell monolayers and the cells washed once with 200 μL PBS. The cells were fixed with cold 80% (v/v) acetone in PBS for 10 min, the fixative removed and the wells air dried. The plate was washed 3 times with wash buffer [PBS containing 1% (v/v) Triton-X100] for 30 s and incubated with 100 μL of a 1:4,000 diluted SARS-CoV-2 nucleocapsid protein monoclonal antibody clone 7E1B (Bioss, Woburn, MA, United States) for 5 min on an orbital shaker (300 rpm) at room temperature and subsequently for 1 h at 37°C. The plates were washed as described and incubated with 100 μL of a 1:10,000 diluted goat anti-mouse IgG (H+L) cross-adsorbed HRP conjugate antibody (Invitrogen, Waltham, MA, United States) for 5 min on an orbital shaker (300 rpm) at room temperature and subsequently for 1 h at 37°C. The plates were washed 5 times with wash buffer for 30 s, followed by 3 washes with deionized water. A 100 μL TMB One substrate (Kementec Solutions, Taastrup, Denmark) equilibrated to room temperature was added to each well and incubated for 15 min at room temperature in the dark. The reaction was stopped by the addition of 100 μL 0.2 M H_2_SO_4_ and the absorbance read at 450 nm with 620 nm as reference on a FluoStar Omega plate reader (BMG Labtech, Offenburg, Germany). At each time point, the average background was calculated from 16 mock infected wells and inoculated wells with an optical density value above 2 × (average background) was considered positive for infection.

### Growth Kinetics

#### Infectious Virus Titre (TCID_50_/mL)

Vero-E6 cells were prepared and inoculated following the protocol described under “Virus titration.” Replicate plates were concurrently prepared from the same cell passage and inoculated with 0.5 log_10_ serially diluted virus from a master virus dilution series. For each virus, the multiplicity of infection (MOI) was 0.01 as determined from the replicate virus titrations at 96 h post-inoculation. SARS-CoV-2 virus infection of inoculated wells was measured using the anti-SARS-CoV-2 nucleocapsid protein ELISA and the TCID_50_/mL at each time point determined using the Reed and Muench method.

#### Genomic and Subgenomic RNA Quantitation

1 × 10^4^ Vero-E6 cells/well were seeded in a 96 well tissue culture plate and incubated overnight at 37°C, 5% CO_2_. On the day of inoculation, the number of cells per well was determined by dissociating cells from quadruplicate wells using TrypLE dissociation media and counting on a haemocytometer. The monolayers were washed once with 200 μL PBS, followed by the addition of 100 μL SARS-CoV-2 viruses diluted with virus diluent to give an approximate MOI of 0.0125 (equates to 2.85 × 10^3^ TCID_50_/mL). Mock infected cells received only infection media. After a 1 h incubation at 37°C, 5% CO_2_, the infected monolayers were washed three times with 200 μL PBS to remove the inoculum, followed by addition of 200 μL growth media (DMEM, 10% fetal calf serum and 1% Penicillin/Streptomycin) to each well. At each indicated time point, the cell culture medium was removed and mixed 1:1 with MagNA Pure Lysis buffer (Roche, Mannheim, Germany); the remaining cells were lysed by adding MagNA Pure Lysis buffer to the wells and incubating for 5 min at room temperature. Each measurement was performed in triplicate. The supernatant and lysed cells were stored at 4°C until extraction on a MagNA Pure.

### Quantitative PCR

SARS-CoV-2 genomic and subgenomic RNA were assayed using real-time reverse transcriptase quantitative PCR. The Luna^®^ Universal Probe One-Step RT-qPCR Kit (New England BioLabs, Ipswich, Massachusetts, United States) was used with primers and probes targeting the envelope (E) gene. For genomic RNA quantitation, the forward primer E_Sarbeco_F1: 5′-ACAGGTACGTTAATAGTTAATAGCGT-3′ and reverse primer E_Sarbeco_R2: 5′-ATATTGCAGCAGTACGCACACA-3′ (400 nM each) were used with the probe E_Sarbeco_P1: 5′-ACACTAGCCATCCTTACTGCGCTTCG-3′ (200 nM). Cycling conditions were 55°C for 10 min, 95°C for 3 min, followed by 45 cycles of 95°C for 15 s and 58°C for 30 s. RNA copy numbers per μL were calculated from a standard curve generated with the Twist Synthetic SARS-CoV-2 RNA Control 1 (Twist Bioscience, San Francisco, California, United States). E gene subgenomic RNA was measured using a forward primer targeting the 5′ untranslated region leader sequence of the genomic RNA sequence, which is incorporated into subgenomic RNAs (sgLeadSARSCoV2-F: 5′-CGATCTCTTGTAGATCTGTTCTC-3′), together with the E_Sarbeco_R2 reverse primer and E_Sarbeco_P1 probe. Cycling conditions were 50°C for 10 min, 95°C for 3 min, followed by 45 cycles of 95°C for 10 s, 56°C for 30 s, and 72°C for 5 s.

### Virus Neutralization

Plasma samples were heat inactivated for 1 h at 56°C before testing in the neutralization assay. A 96-well tissue culture plate was seeded with 1 × 10^4^ Vero-E6 cells per well. The following day, a twofold dilution series of heat-inactivated convalescent plasma was prepared in virus diluent. Fifty μL diluted serum/plasma was mixed with 50 μL virus diluent containing 300 × TCID_50_ SARS-CoV-2 virus, as calculated from the 96 h virus titrations and incubated for 1 h at 37°C, 5% CO_2_. The cell monolayer was washed once with 200 μL PBS and the serum-virus mixture added to the wells. The inoculated cells were incubated at 37°C, 5% CO_2_ for 24 h. Viral growth was measured using the anti-nucleocapsid ELISA as described. At 24 h post-inoculation, the ELISA measurements directly correlate with the level of virus, as observed for virus titrations measured at 24 h (Spearman *r* > 0.950 for different strains, *P* < 0.0001). Included on each microneutralization plate were quadruplicate virus control wells inoculated with 300 × TCID_50_ of each SARS-CoV-2 virus without serum and quadruplicate cell control wells with mock infected cells. A 50% virus inhibition cut-off was calculated for each virus using the virus control wells specific for that virus with the following equation (average OD of virus control wells + average OD of cell control wells)/2. To account for the difference in growth kinetics observed at 24 h post-inoculation, the virus neutralization was determined relative to the maximum signal for each virus as observed for the appropriate virus control wells. A four parameter logistic regression curve was generated for each serum titration and the 50% neutralization titre interpolated from the virus-specific cut-off. To minimize inter-assay variation, each serum/plasma sample was tested against both virus strains on a single 96-well tissue culture plate. On each plate, the serum sample was tested in triplicate with both viruses and the neutralization titre calculated from virus and cell control wells included on that plate.

### Serum/Plasma Samples

An initial screening panel of nine convalescent plasma were selected from persons living in the Capital Region in southern Denmark, who were geographically separated from the mink outbreaks in Jutland in northern Denmark, and had a documented SARS-CoV-2 infection at the beginning of the Danish epidemic before the mink outbreaks occurred. In the initial study (Lassaunière et al., 2020), nine convalescent samples were tested in duplicate (data not shown). For plasma samples with sufficient material (8 of 9 samples), an independent replicate analysis with triplicate determinants was performed and is presented in the present study. Sera with previously determined low (*N* = 3), intermediate (*N* = 3) and high (*N* = 2) neutralization titres against an early pandemic SARS-CoV-2 virus in the aforementioned neutralization test were used. Each plasma sample represented a different donor. Additional serum and plasma samples (*N* = 36) with a range of neutralization titres against an early SARS-CoV-2 pandemic strain were subsequently tested, each in triplicate. Convalescent serum/plasma samples were from Danish blood donors and volunteers recruited at Hvidovre Hospital, Denmark.

### Biosafety and Security

All experiments were performed in a Biosafety Level 3 (BSL3) laboratory according to standard biosecurity and institutional safety procedures. Biological material used in this study are contained in an access controlled building and BSL3 laboratory with limited access by appropriately trained personnel. Following each experiment, material containing live virus was inactivated with 2% Virkon S followed by autoclaving.

## Data Availability Statement

The datasets presented in this study can be found in online repositories. The names of the repository/repositories and accession number(s) can be found below: https://www.gisaid.org/, EPI_ISL_616802.

## Ethics Statement

The studies involving human participants were reviewed and approved by the Danish Regional Committee on Health Research Ethics. The patients/participants provided their written informed consent to participate in this study.

## Author Contributions

RL, AFr, and CP performed the experiments and data analysis. RL drafted the manuscript and designed the figures. JF, MR, AA-N, TR, and LL directed the sequencing and phylogenetic analyses. AU, AW, SB, JB, NW, SS, and BJ collected convalescent patient samples. AB and AFo designed and directed the project. All authors critically reviewed the manuscript.

## Conflict of Interest

The authors declare that the research was conducted in the absence of any commercial or financial relationships that could be construed as a potential conflict of interest.
