# Supplementary material for: In vitro Characterization of Fitness and Convalescent Antibody Neutralization of SARS-CoV-2 Cluster 5 Variant Emerging in Mink at Danish Farms
Source: Front Microbiol. 2021 Jun 25;12:698944. doi: 10.3389/fmicb.2021.698944 (PMC8267889; doi:10.3389/fmicb.2021.698944)
Supplement: Supplementary file 1 [file Data_Sheet_1.docx]

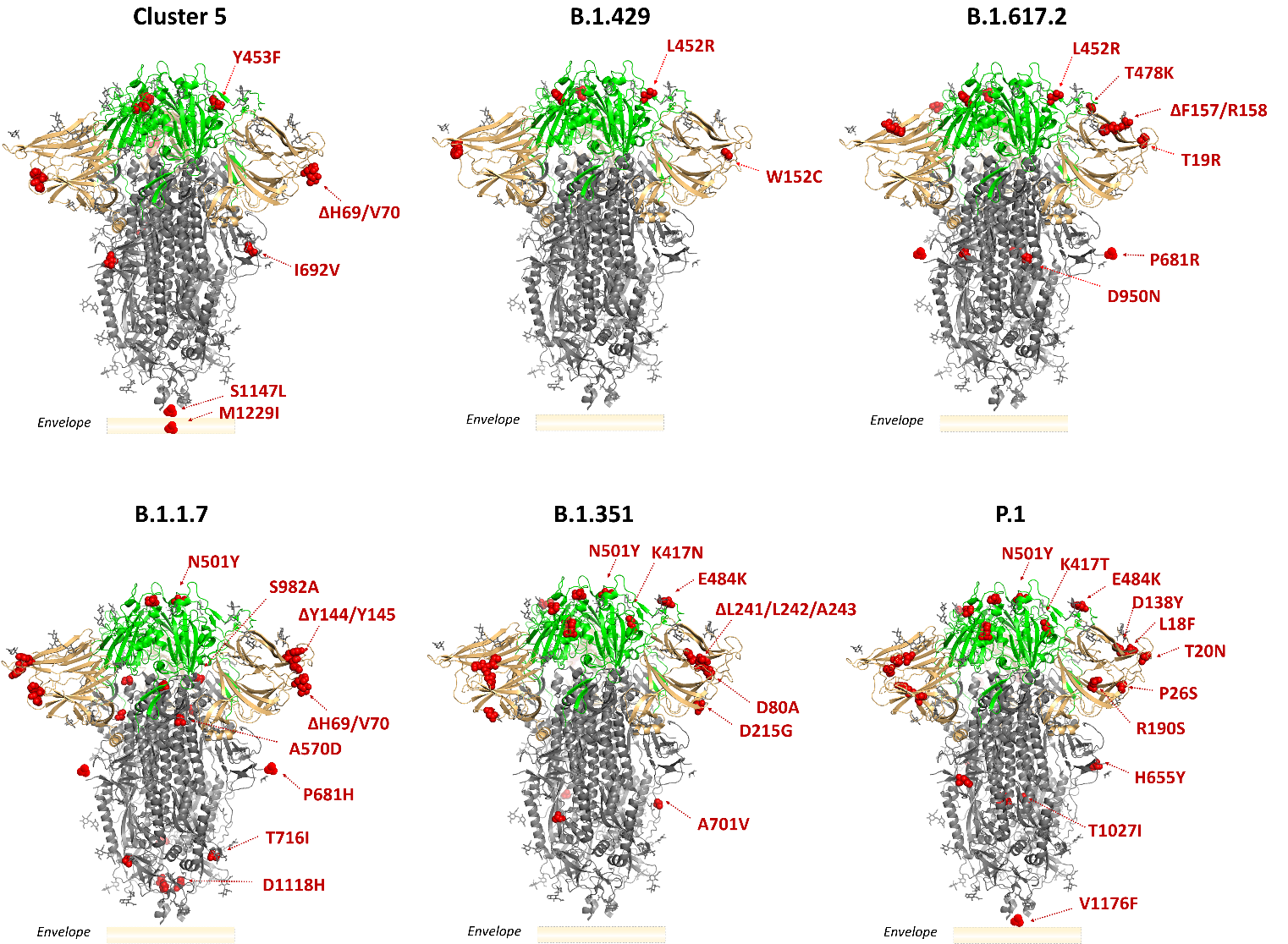


**Supplementary Figure 1.** **Amino acid substitutions and deletions in the spike protein sequences of** **SARS-CoV-2 Cluster 5 relative to circulating variants of concern (VOCs).** Presented VOCs are as defined by the Centers for Disease Control and Prevention USA and European Centers for Disease Control on 25 May 2021. Only amino acid changes present in >50% of lineage-specific sequences in GISAID are presented. Red spheres indicate the position of amino acid changes or deletions on a closed pre-fusion spike trimer [PDB: 6ZGE]. The receptor binding domain is in green, the N-terminal domain in beige, and the S2 domain in grey.
